# Cell wall metabolism and hexose allocation contribute to biomass accumulation in high yielding extreme segregants of a *Saccharum* interspecific F2 population

**DOI:** 10.1186/s12864-017-4158-8

**Published:** 2017-10-11

**Authors:** Ching Man Wai, Jisen Zhang, Tyler C. Jones, Chifumi Nagai, Ray Ming

**Affiliations:** 1 FAFU and UIUC-SIB Joint Center for Genomics and Biotechnology, Fujian Provincial Key Laboratory of Haixia Applied Plant Systems Biology, Key Laboratory of Genetics, Breeding and Multiple Utilization of Corps, Ministry of Education, Fujian Agriculture and Forestry University, Fuzhou, Fujian 350002 China; 2 Department of Plant Biology, University of Illinois at Urbana-Champaign, Urbana, IL USA; 3 Hawaii Agriculture Research Center, Kunia, HI USA

**Keywords:** *Saccharum*, Biomass, Transcriptome, Cell wall metabolism, Hexose allocation

## Abstract

**Background:**

Sugarcane is an emerging dual-purpose biofuel crop for energy and sugar production, owing to its rapid growth rate, high sucrose storage in the stems, and high lignocellulosic yield. It has the highest biomass production reaching 1.9 billion tonnes in 2014 worldwide.

**Results:**

To improve sugarcane biomass accumulation, we developed an interspecific cross between *Saccharum officinarum* ‘LA Purple’ and *Saccharum robustum* ‘MOL5829’. Selected F1 individuals were self-pollinated to generate a transgressive F2 population with a wide range of biomass yield. Leaf and stem internodes of fourteen high biomass and eight low biomass F2 extreme segregants were used for RNA-seq to decipher the molecular mechanism of rapid plant growth and dry weight accumulation. Gene Ontology terms involved in cell wall metabolism and carbohydrate catabolism were enriched among 3274 differentially expressed genes between high and low biomass groups. Up-regulation of cellulose metabolism, pectin degradation and lignin biosynthesis genes were observed in the high biomass group, in conjunction with higher transcript levels of callose metabolic genes and the cell wall loosening enzyme expansin. Furthermore, UDP-glucose biosynthesis and sucrose conversion genes were differentially expressed between the two groups. A positive correlation between stem glucose, but not sucrose, levels and dry weight was detected.

**Conclusions:**

We thus postulated that the high biomass sugarcane plants rapidly convert sucrose to UDP-glucose, which is the building block of cell wall polymers and callose, in order to maintain the rapid plant growth. The gene interaction of cell wall metabolism, hexose allocation and cell division contributes to biomass yield.

**Electronic supplementary material:**

The online version of this article (10.1186/s12864-017-4158-8) contains supplementary material, which is available to authorized users.

## Background

Due to the limited supply of non-renewable energy such as fossil fuels and coal, the need to develop alternative sources of energy is increasing as the world population increases. Cellulosic biofuel, which is bioenergy derived from the plant cell wall, is one of the main potential sources of renewable energy. Plant-based bioenergy generates environmentally friendly energy with 7% to up to 4-fold less greenhouse gas emissions, depends on the feed stocks and form of energy produced [1, 2]. Unlike the plant starch-derived bioethanol, cellulosic materials mainly come from the non-consumable parts of plants, and thus would not compete with food production and inflate food prices. The renewable energy generated from bioethanol and lignocellulosic feedstock has been increasing gradually in recent years but the supply from lignocellulosic materials lag behind the proposed expectation from the US renewable fuel standard program. According to the renewable fuel standard II (RFS2), in 2015, oil companies should have produced 77.6 billion liters of renewable energy of which 11 billion liters should have come from cellulosic biofuel (RFS). However, only 538 million liters were generated from cellulosic materials in 2015 (US EPA, 2015). Furthermore, by 2022, 61 billion liters of fuel for the US transportation industry should be generated from lignocellulosic feedstock [3]. Thus, a rapid improvement in biofuel crop breeding and facilities for energy extraction is necessary to meet the expected supply in 2022.

C_4_ grasses, including maize, sorghum, switchgrass, sugarcane and miscanthus, are the best candidate species for cellulosic biofuel production due to their high biomass yield, rapid growth rate and better adaptation to marginal soils. Among sugarcane, sorghum and maize, sugarcane has the highest lignocellulose yield and lowest water requirement per dry matter yield [4]. Sugarcane is the largest crop in terms of weight with 1.9 billion tonnes produced in 2014 (FAOSTAT, 2014). The bagasse, the dry pulp remaining after juice extraction, is used as fuel. In 2009, sugarcane bagasse produced 15% of the total energy consumed in Brazil [5]. In the past, extensive breeding programs were undertaken to select high sucrose content cultivars for sugar production. Recently, energy cane with high fiber content and dry matter yield gained the attraction of researchers and breeders for biofuel production. The successful implementation of sugarcane as a source of lignocellulosic biofuel relies on three factors: 1) High dry mass yield per hectare; 2) High percentage of soluble sugar from dry biomass; 3) Cell wall components content with high digestibility.

To improve the yield and energy conversion efficiency of biofuel crops, cell wall components were genetically engineered. Successful improvement of both biomass yield and digestibility was achieved in poplar by engineering pectin methyltransferase and galacturonosyl transferase expression levels [6, 7]. Transgenic switchgrass overexpressing the R2R3-MYB transcription factor PvMYB4 showed up to 50% decrease in lignin content with up to 63% increase in biomass [8]. However, the majority of engineering attempts of cell wall components negatively impacted plant growth [9]. For example, transgenic sugarcane plants with RNAi and TALEN down-regulating caffeic acid 3-O-methyltransferase, showed reduced lignin content, which can improve recalcitrance [10, 11]. However, up to 35% biomass reduction with decreased stalk diameter was observed in transgenic lines with high lignin reduction [11]. Affected growth with reduced biomass was also reported in other transgenic plant systems with altered lignin synthesis genes such as caffeoyl shikimate esterase, cinnamyl alcohol dehydrogenase, and laccase [12–14]. Therefore, a complete understanding of cell wall metabolism and gene interaction is necessary for successful genetic engineering. Genome-wide transcriptomic studies help dissecting the relationship between photosynthesis, cell division, cell wall and sugar metabolism, as all these pathways are interconnected. In addition, different enzymatic treatments currently exist for various recalcitrant modified plants, it is therefore important to understand the cell wall metabolism mechanism leading to cell wall composition variation.

In addition to genetic engineering, it was proposed that high biomass sugarcane can be achieved by crossing commercial sugarcane *Saccharum officinarum* with fibrous *Saccharum spontaneum* and *Saccharum robustum* for high fiber and sugar content variety development [15]. Recent exploitation of 1002 accessions from the World Collection of Sugarcane and Related Grasses in Florida suggests a high genetic diversity of the *Saccharum* species with close genetic distance found between *S. officinarum* and *S.robustum* [16]. Thus, we developed a F1 population from the interspecific cross between *S. officinarum* ‘LA Purple’ and *S. robustum* ‘MOL5829’ to investigate the effect of *S. robustum* on biomass improvement. To further enhance the yield, the extreme F1 segregants in the highest and lowest biomass groups (9–10 progenies from each group) were crossed within the group to produce a F2 population, and the dry matter biomass of the F2 progenies was measured for 3 years. With the availability of extreme performing F2 progenies with similar genetic backgrounds, we were able to study the gene expression difference between two groups using RNA-seq, which enhanced our knowledge on the molecular mechanism of biomass accumulation in *Saccharum* interspecific cross. Simultaneously, the high yield F2 progenies can be used in breeding programs to develop sugarcane cultivars with both high biomass and sucrose content as a dual-purpose biofuel crop for sugar and energy production.

## Methods

### Plant materials

An interspecific cross was produced between *Saccharum officinarum* ‘LA Purple’ (2n = 8× = 80) and *Saccharum robustum* ‘MOL5829’ (2n = 8× = 80) [17]. Ninety-eight F1 progenies were field tested for stem diameter, stem weight, sugar and fiver content. Then, twenty F1 progenies showing different phenotypes of stem diameter, stem weight, and fiber content were chosen for self-fertilization to generate a F2 population with transgressive biomass traits between 2007 and 2011 at Hawaii Agriculture Research Center Maunawili station, Oahu, Hawaii. Two hundred and seventy-four F2 progenies were grown at various stations in Hawaii Commercial and Sugar Company, Maui for field testing and 120 F2 plants with extreme biomass yield were selected. The biomass performance of these 120 plants was further confirmed in field trial carried out at Hawaii Agriculture Research Center Kunia station, Oahu, Hawaii. Out of these 120 F2 plants, 22 were used in this RNA-seq study. These 22 F2 progenies were divided into two groups based on their three year average biomass measurement: the F2 plants above the 75th percentile of a three year average dry weight were classed as the high biomass group (14 F2 progenies), whereas F2 plants below the 25th percentile of the three year average dry weight were grouped in the low biomass group (8 F2 progenies). All plants materials used for RNA-seq and sugar content studies were grown at the Hawaii Agriculture Research Center Kunia substation (Hawaii, USA), and Texas A&M AgriLife Research and Extension Center at Weslaco (Texas, USA).

### Field biomass measurements

To collect biomass measurements of the 120 F2 progenies, the total dry weight (in tonnes per hectares in a 12-month growth period) from 0.46-square meter plots was measured for three years between 2010 and 2012. The field trial of 2010 was carried out in Maui, Hawaii while the other two field trials were carried out in Kunia, Hawaii. Both the 2010 and 2011 field measurements used the first-year crop while the 2012 measurement used the ratoon crop planted in 2011. For 2010 field testing, only 1 plot was planted for each genotype while for 2011 and 2012 field testing, 3 replicates plot were planted per genotype based on randomized block design. Only the 22 F2 progenies used in this study was reported.

### RNA extraction and library construction

The first dewlap leaf, stem internodes from 3rd, 9th, and 15th node (internode at the first dew lap leaf was numbered as first internode) were collected from 8-month old F2 progenies and frozen in liquid nitrogen immediately. Eight low biomass and four high biomass individuals were collected from Texas station while 10 high biomass individuals were collected from Hawaii. For internode, the number was counted starting from the first dew lap leaf and ~1.5 cm above and below the node were collected. Tissues were then stored at −80 °C until ground into powder. 100 mg of ground tissue was used for RNA extraction using the Omega Bio-tek E.Z.N.A.® Plant RNA kit (Omega Bio-tek, #R6827–02). The three internode samples were pooled to form equal weight samples prior to RNA extraction. The leaf and internode RNA were treated to remove genomic DNA using the Ambion DNA-*free*™ DNA removal kit (Life Technolgies, #AM1906) and then quantified by NanoDrop 2000 (ThermoFisherScientific Inc.). One μg of leaf RNA was mixed with one μg of internode RNA and then subjected to Illumina RNAseq library construction using the Illumina stranded mRNA sample prep LT kit (Illumina, #RS-122-2101), according to the manufacturer’s instructions. The multiplexed libraries were pooled and sequenced by HiSeq2500 paired end 150 nt. The raw data can be accessed at NCBI BioProject ID PRJNA347369 and Short Read Archive under SRR5223352 - SRR5223361.

### Sequence read alignment and differential expression analysis

Paired-end read alignment was carried out by NovoAlign CS (http://www.novocraft.com/documentation/novoaligncs-3/) against *Sorghum bicolor* v2.1 gene annotation with longest transcripts only (Sbicolor_255_v2.1_transcript_primaryTranscriptOnly.fa.gz) downloaded from Phytozome v10 (http://genome.jgi.doe.gov/pages/dynamicOrganismDownload.jsf?organism=PhytozomeV10), using default parameters and 3′ adapter trimming. Unambiguous reads aligned to each gene were counted using htseq-count version 0.6.1p2 [18]. Differential gene expression between high biomass group (*n* = 14) and low biomass group (*n* = 8) F2 progenies was analyzed using EdgeR, a R bioconductor package [19]. Genes with more than 2-fold change (i.e. log_2_FC ≥ 1) with *p*-value ≤0.01 and false discovery rate (FDR) ≤ 0.05 were classed as differentially expressed (DE) genes in this study.

### Sugar content measurement of F2 progenies

The ground leaf and pooled internode tissues (i.e. stem) used for RNA-seq studies (see above) were also used to measure soluble glucose and sucrose levels. Sugar content was measured from 100 mg of ground tissue extracted in 1 mL of 80% HEPES buffered ethanol (pH 7.8), using Glucose, and Sucrose assay kits, respectively (Sigma-Aldrich, #GAHK20, #SCA20) according to the manufacturer’s protocols. The leaf and stem sugar content were then expressed as glucose (or sucrose) concentration of fresh weight (μg/mg).

## Results

### Differentially expressed genes between high and low biomass sugarcane plants are enriched in cell wall metabolism

To understand the molecular mechanism of fast-growing sugarcane with high dry weight accumulation, we developed an interspecific F2 population from the cross between *Saccharum officinarum* ‘LA Purple’ and *Saccharum robustum* ‘MOL5829’. The resulting F1 sugarcanes were phenotyped for biomass traits including dry weight, stalk diameter and stalk number. Extreme F1 segregants from the top ten highest (“high biomass group”) and the bottom ten lowest (“low biomass group”) dry weight were self-pollinated to produce F2 progenies. We then selected 14 F2 progenies from the high biomass group and eight F2 progenies from the low biomass group for RNA-seq study, carbohydrate and dry weight measurements. The dry weight (in metric ton per hectare per 12-month) of F2 progenies grown on 0.46-square meter plots was measured in 2010–2012 (Table 1).

**Table 1:** Dry weight of ‘LA Purple’ x ‘MOL5829’ F2 progenies during 2010–2012

F2 progeny ID	PCA ID	Dry weight (MT/ha/12 month)			Average 3-year dry weight
		2010	2011	2012	
High biomass group					
09–9183	4	70.6	113.9	135.0	106.5
09–9006	5	63.8	118.2	136.7	106.2
09–9178	6	90.6	125.7	91.3	102.5
09–9078	8	20.9	108.2	159.3	96.1
09–9019	9	60.8	112.6	107.9	93.8
09–9126	10	27.3	124.9	120.0	90.7
09–9169	12	35.4	124.7	105.3	88.4
09–9009	13	41.8	110.6	110.2	87.5
09–9053	14	23.5	116.9	120.6	87.0
09–9117	17	23.3	124.2	104.6	84.1
09–9001	18	42.3	116.9	89.8	83.0
09–9251	20	29.0	79.5	130.5	79.7
09–9010	22	42.0	130.6	63.7	78.8
09–9148	23	35.3	106.6	94.2	78.7
Low biomass group					
09–9074	90	15.7	65.4	29.9	37.0
09–9190	93	14.7	65.2	29.3	36.4
09–9069	97	13.1	53.1	39.7	35.3
09–9163	98	12.3	52.1	38.6	34.3
09–9065	104	25.1	55.1	12.2	30.8
09–9197	106	30.8	37.2	14.5	27.5
09–9095	109	18.3	34.3	N/A	26.3^a^
09–9246	112	6.7	28.2	18.9	18.0
LA Purple	N/A	81.4	51.6	66.5	66.5

Sugarcanes were grown in 5 ft plot at Hawaii Agriculture Research Center Maui and Kunia substations in 2010 and 2011–2012, respectively. The dry weight (in metric ton per hectare per 12-month) of each F2 progeny were measured. The 2010 and 2011 crops are newly planted seed pieces while 2012 plants are ratoon crops

*N/A* not available, *PCA ID* ID number used in Principal component analysis (PCA) plot

^a^Average yield from 2 year measurement

**Fig. 1:**
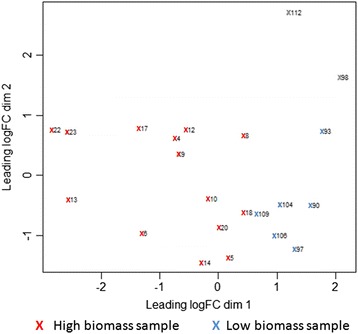
Principal component analysis (PCA) of transcriptomic expression of high and low biomass F2 sugarcanes. RNA-seq expression of all genes of high biomass (red cross) and low biomass (blue cross) sugarcane individuals were analyzed by PCA. Sample ID is listed in Table 1. High and low biomass samples are labeled as (red X) and (blue X) cross, respectively

For the transcriptomic study, equal amounts of leaf, 3rd, 6th, and 9th internodes (see method for definition) RNA of each F2 progeny were mixed for library construction and sequencing. Since a well-assembled *Saccharum* reference genome is not available, the RNA-seq reads were aligned to the *Sorghum bicolor* annotated gene sequences; GO and metabolic pathway analyses were based on sorghum gene models. To study the variation between F2 progenies, whole genome transcript expression of each individual was analyzed by Principle component analysis (PCA, Fig. 1). The high and low biomass group samples did not cluster tightly among individuals within the same group. This is likely due to the polyploid genome causing wide genetic diversity of the F2 population. These two groups could however be mainly separated by first component, with the second component having very little effect. Among the 33,032 annotated sorghum genes, 3274 genes were differentially expressed (log2 fold change ≥ |1|, *p-value* ≤ 0.01 and FDR ≤ 0.05) between high and low biomass group and 2475 and 799 genes are up- and down-regulated in the high biomass group, respectively.

**Fig. 2:**
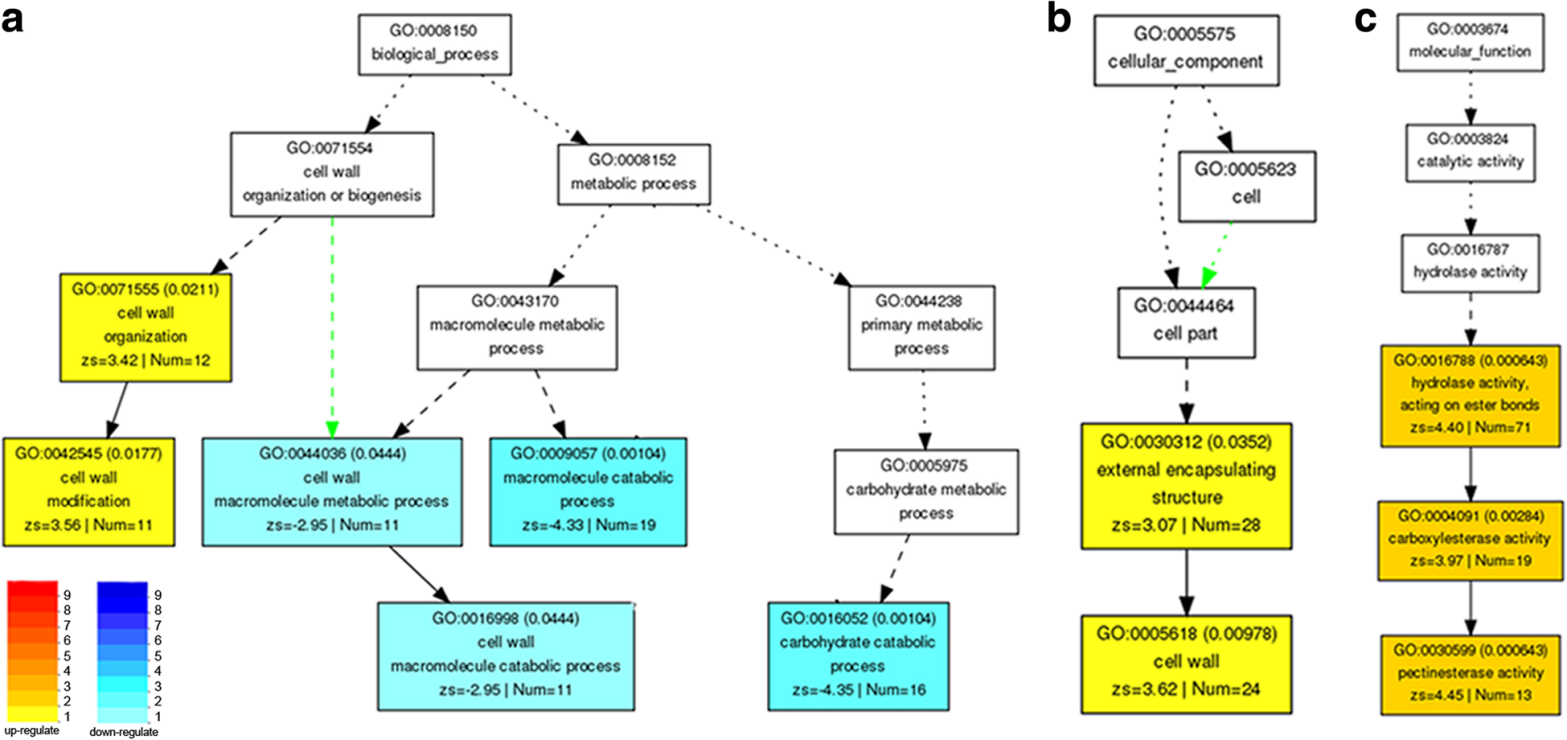
GO enrichment analysis of differentially expressed genes between extreme segregated sugarcanes. 3274 differentially expressed genes between high and low biomass group were tested for GO enrichment based on pathways involved in (**a**) biological function, (**b**) cellular process and (**c**) molecular process

To begin to understand the pathways that differentially expressed genes are involve in, we performed the Gene Ontology (GO) term enrichment test on these 3274 differentially expressed genes with the web-based tool AgriGO based on three categories: biological function, cellular process and molecular process. In the high biomass group, there is an enrichment of up-regulated genes related to cell wall modification (*p*-value =0.018) and down-regulated genes involved in cell wall macromolecule (*p*-value =0.044) and carbohydrate catabolic process (*p*-value =0.001) (Fig. 2a). Furthermore, GO terms involved in cell wall cellular process (*p*-value =0.010) (Fig. 2b) and pectinesterase activity (*p*-value =0.001) (Fig. 2c) were also enriched in up-regulated genes. These results suggested that cell wall metabolic genes, especially pectinesterase, are highly expressed in the high biomass group, whereas genes involved in cell wall catabolism are suppressed. This led us to hypothesize that the cell wall anabolism and catabolism are among the key regulators of biomass accumulation in this F2 population.

### High transcript expression of cell wall and cell plate metabolism genes were detected in high biomass F2 sugarcane progenies

The over-representation of cell wall related genes in the GO enrichment analysis among differentially expressed genes led us to study the genes involved in cell wall metabolism and cell growth in detail. The cell wall is mainly composed of cellulose, hemicellulose, lignin, pectin and some structural proteins. The cell wall composition varies between cell type, developmental stage within plants and even across species. Little is known about the cell wall composition in sugarcane [20] and very few genes involved in cell wall metabolism are characterized even in the Poaceae family of grasses*.*

Cellulose is the most abundant biopolymer on earth and represents 42–46% (*w*/w) of sugarcane cell wall components [21]. It is composed of UDP-α-D-glucose through the catalysis by cellulose synthase, and degraded by cellulase. Among 25 annotated sorghum cellulose synthase and 28 cellulase genes, 5 (20%) and 7 (25%) of them, respectively, were up-regulated in high biomass sugarcane and no down-regulation was observed (Table 2). Hemicellulose is the second major cell wall component and the number of differentially expressed genes involved in xylan biosynthesis and catabolism was less abundant than that observed in cellulose metabolism. In total, four up-regulated xylan synthases, three up-regulated and one down-regulated xylanase were found between two groups of sugarcanes.

**Table 2:**
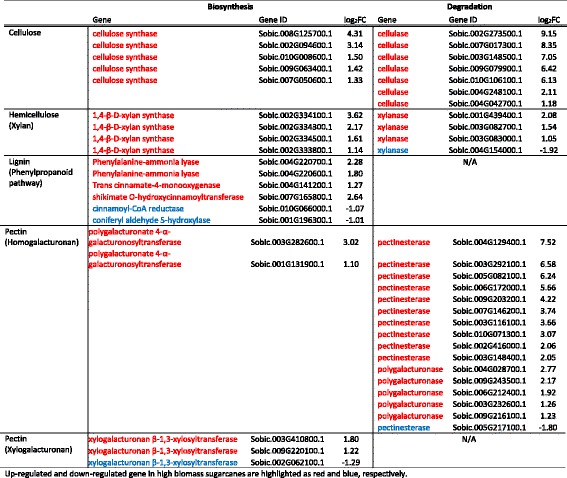
Differentially expressed genes involved in cell wall metabolism pathway

Up-regulated and down-regulated gene in high biomass sugarcanes are highlighted as red and blue, respectively

Pectin, a complex polysaccharide mainly only found in sugarcane primary cell wall, is comprised of homogalaturonan, xylogalacturonan and rhamnogalacturonan I and II. The catabolism of xylogalacturonan and metabolism of rhamnogalacturonan are not characterized and thus not discussed in this study. For homogalaturonan and xylogalacturonan biosynthesis, two polygalacturonate 4-α-galacturonosyltransferase and xylogalacturonan β-1,3-xylosyltransferase genes were up-regulated while one xylogalacturonan β-1,3-xylosyltransferase gene was down-regulated (Table 2). We observed a large number of differentially expressed genes involved in homogalacturonan breakdown. Among 56 annotated pectinesterase genes in the sorghum genome, 10 were up-regulated while 1 was down-regulated in high biomass sugarcanes. This enzyme family is over-represented in GO enrichment analysis (Fig. 1). In addition, 5 out of 27 polygalacturonase genes had higher levels of expression in the high biomass group. This indicates that the pectin polymer breaks down rapidly for recycling in the high biomass sugarcanes.

Unlike pectin, lignin is mostly found in the secondary cell wall, and is the second most abundant polymer on earth after cellulose. Lignin is comprised of amorphous polymers called monolignols, which are synthesized from phenylalanine through the phenylpropanoid pathway. The monolignols are then transported to the cell wall and polymerized by radical coupling via laccase and peroxidases. There was a minor difference in gene expression levels between high and low biomass sugarcanes in the phenylpropanoid biosynthesis genes, with four up-regulated and two down-regulated genes (Table 2). However, 11 laccase genes were expressed differently between the two groups, 8 were up-regulated and 3 were down-regulated (Table 3). These results suggest that the biosynthesis of lignin precursors does not contribute to the observed biomass difference between two groups, but polymerization of monolignols through laccase might play an important role in secondary cell wall synthesis in the high biomass group.

**Table 3:**
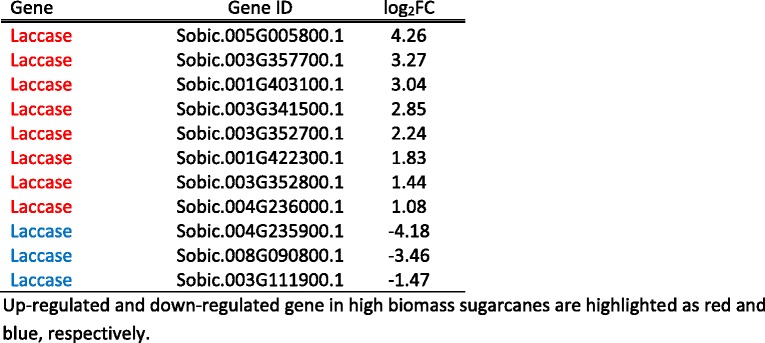
List of differentially expressed laccase genes

Up-regulated and down-regulated gene in high biomass sugarcanes are highlighted as red and blue, respectively

Plants can grow by either of two mechanisms - increasing cell number through cell division, and/or enlarging cell size by loosening the cell wall and intaking solutes. Both mechanisms require the reconstruction of cell wall structures. In our study, the high transcript levels of expression of cellulose synthesis and degradation genes together with the rapid degradation of the pectin polymer reflects the dynamic cell wall components turnover in high biomass sugarcanes. We thus speculated the sugarcanes in the high biomass group accumulate higher total dry mass because they have faster cell division rates. However, it is difficult to measure the rate in vivo without cutting the whole plantlet. Instead, we surveyed key genes involved in cell plate formation (e.g. callose synthase), and cell wall loosening (e.g. expansin) providing a snapshot of the cell division and elongation activities.

During cell division, the cell plate formed between two daughter cells is composed of callose, a β-1,3 linked glucan. It is synthesized in the cell wall by callose synthase and degraded by β-1,3-glucosidase. In the high biomass group, 3 out of 12 annotated callose synthase genes were up-regulated together with 15 up-regulated and 5 down-regulated β-1,3-glucosidase genes (Table 4). These 20 differentially expressed β-1,3-glucosidase genes account for one-third of the 60 annotated genes. Concurrently, we observed transcript up-regulation of four expansin A and four expansin B genes, and down-regulation of only one expansin A gene (Table 5). These data supported our hypothesis that high biomass sugarcanes undergo rapid cell division and thus have higher demands for cell wall polymer biosynthesis and degradation, which in turn lead to increases in total biomass.

**Table 4:**
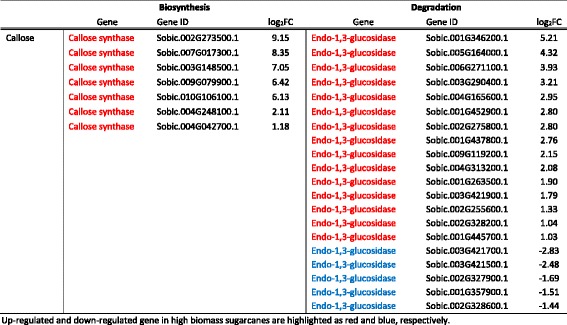
Differentially expressed genes involved in callose metabolism

Up-regulated and down-regulated gene in high biomass sugarcanes are highlighted as red and blue, respectively

**Table 5:**
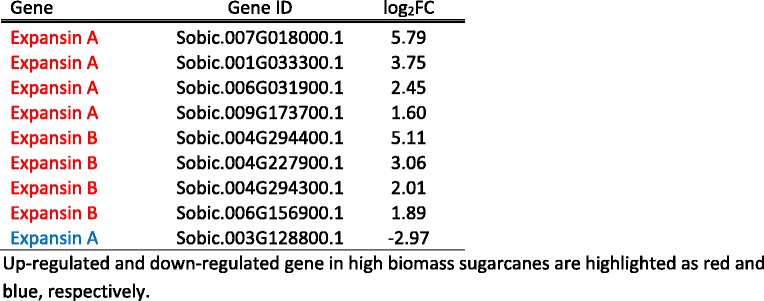
List of differentially expressed expansin family genes

Up-regulated and down-regulated gene in high biomass sugarcanes are highlighted as red and blue, respectively

### Glucose and sucrose metabolism and transport are altered in high biomass sugarcane

Surveying the cell wall metabolism pathways clearly showed that a large portion of differentially expressed genes between high and low biomass sugarcanes are related to cellulose metabolism, monolignol polymerization, pectin degradation, callose metabolism and cell wall loosening. The major building block of cell wall polymers is UDP-glucose, which is also one of the intermediates in the sucrose biosynthesis pathway. This led us to raise questions about (1) photosynthetic gene expression increases for higher photosynthate production and accumulation; (2) glucose precursors that are allocated to cell wall metabolism from sucrose biosynthesis and storage in stem parenchyma cells.

To address the first question, we examined transcript expression of genes involved in carbon fixation, the Calvin cycle and gluconeogenesis, and only observed differential expression of one gene in carbon fixation and the Calvin cycle, and four genes in gluconeogenesis between high and low biomass group (Additional file 1: Table S1). This suggested that photosynthesis related transcript expression does not contribute to the biomass accumulation. However, we cannot rule out the possibility that there could be photosynthetic rate differences between the two groups without measuring carbon fixation and energy conversion rate, or that biomass accumulation is associated with translational regulation of photosynthetic genes.

**Fig. 3:**
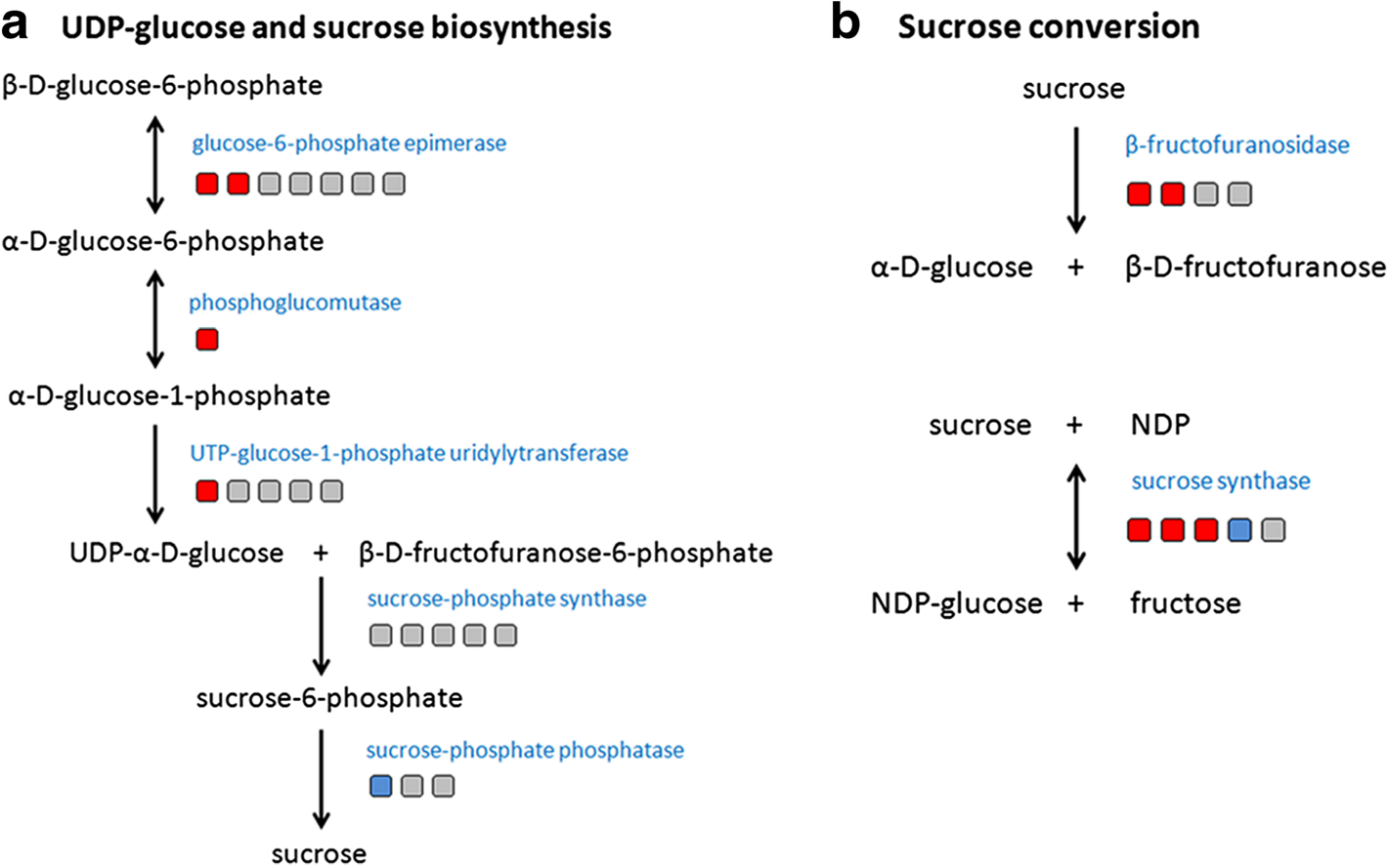
Differentially expressed genes between high and low biomass groups involved in hexose metabolism and conversion pathways. Differentially expressed genes involved in (**a**) UDP-glucose and sucrose biosynthesis, and (**b**) sucrose conversion pathway was labeled. Each box represents a gene copy whereas up-regulated, down-regulated, and no difference genes are colored as red, blue, and grey, respectively

Next, we examined whether there is an increase of hexose production or conversion of stored sucrose to hexose in the high biomass group. When we surveyed genes involved in sucrose and UDP-glucose metabolism, we observed a dynamic change of gene expression in these pathways. Specifically, four genes involved in the conversion of β-D-glucose-6-phosphate to UDP-α-D-glucose were up-regulated in high biomass sugarcane (Fig. 3a). β-D-glucose-6-phosphate is the product of gluconeogenesis and its conversion to UDP-α-D-glucose is an important metabolic step since UDP-α-D-glucose is the major component in both cellulose and sucrose biosynthesis. For the latter, UDP-glucose can then be converted to sucrose by sucrose phosphate phosphatase, where one of three homologs were down-regulated in high biomass sugarcane plants (Fig. 3b). Sucrose synthase (SuSy) is another enzyme that can convert UDP-glucose to sucrose. Although this reaction is reversible, SuSy expression is negatively correlated to sucrose content and positively correlated to hexose content in sugarcane [22]. Among the five SuSy homologs, three were up-regulated while one was down-regulated in the two sugarcane groups. Furthermore, two β-fructofuranosidase genes, which irreversible convert sucrose to D-glucose and D-fructofuranose, were expressed strongly in the high biomass group. These results indicated that in high biomass sugarcane, the conversion between sucrose, glucose and fructose are very active. Based on the down-regulated sucrose phosphate phosphatase and up-regulated β-fructofuranosidase observed transcript expression, we hypothesized that storage sucrose is mobilized to glucose and fructose for energy and cell wall production in the high biomass group in order to support rapid cell division.

**Fig. 4:**
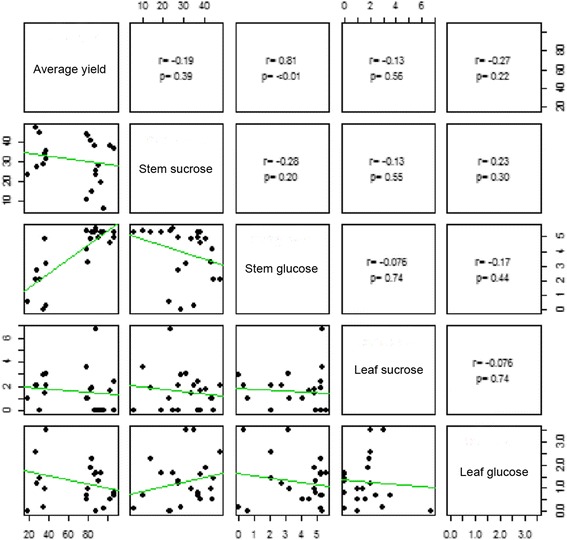
Pearson correlation of average dry mass yield, sucrose and glucose level in stem and leaf of 22 interspecific *Sacchrum* F2 progenies. Three-year average dry mass (in metric ton per hectare per 12 month), soluble sucrose and glucose content in stem and leaf (in μg per mg fresh weigh) were measured for 12 high biomass and 8 lower biomass sugarcanes. The correlation (r) with *p-*value (p) was calculated for pairwise comparison

To support this hypothesis, we measured the glucose and sucrose content in the leaf and stem tissues of all 22 F2 progenies from this study, to test if there is any correlation with the 3-year average dry matter biomass. The ground tissue used in sugar measurement was the same tissue used for RNA-seq analysis. Interestingly, there was a strong positive correlation between stem glucose level and average yield (*r* = 0.81, *p-value* < 0.01) but no correlation between stem sucrose level, leaf glucose level, leaf sucrose level with biomass yield (Fig. 4). These physiological measurements complement the transcript expression differences of sucrose conversion and UDP-glucose metabolism genes between two groups, suggesting glucose accumulates in stems to support rapid cell growth in the high biomass group. However, we cannot rule out the possibilities of carbohydrate partitioning between leaf and root and respiration rate also affecting the photosynthase accumulation and thus, biomass production.

**Fig. 5:**
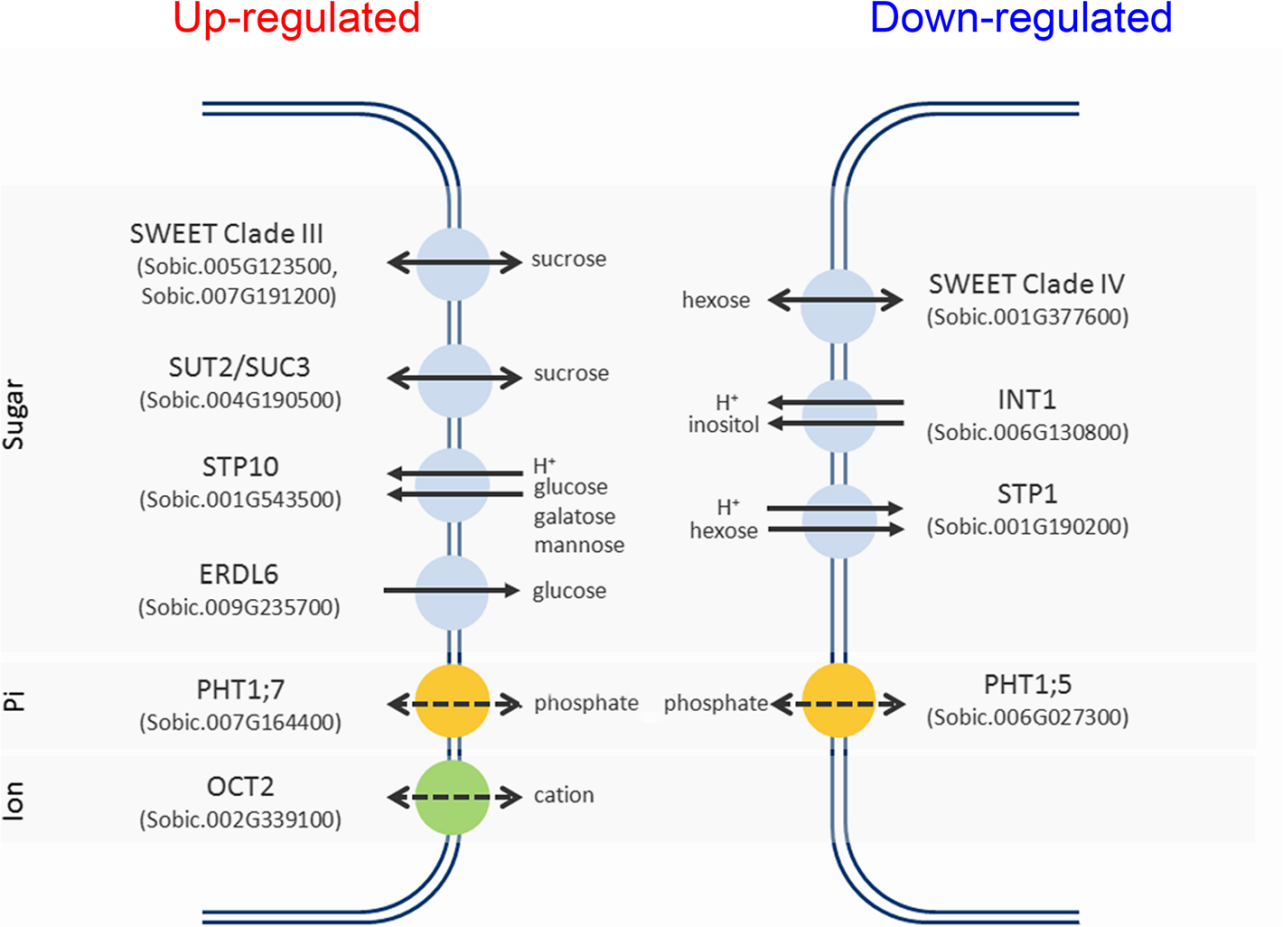
Transporters with differential expression between high and low biomass sugarcanes. Up-regulated transporters are listed on left panel while down-regulated one are listed on right panel. Transporters are categorized into three groups – sugar, phosphate and ion transporters and corresponding sorghum gene ID is listed. The transporters action mode and binding affinity are predicted based on its arabidopsis homolog

In addition to sugar metabolism, transport of hexoses between cell compartments was also affected in high biomass sugarcane. Two SWEET clade III transporters and sucrose transporter 2 (SUT2/SUC3), which mainly transport sucrose, were strongly expressed. The vacuolar glucose exporter ERDL6 and glucose, galactose and mannose importer sugar transport protein 10 (STP10) were also up-regulated (Fig. 5). On the other hand, a SWEET clade IV hexose transporter, myo-inositol exporter inositol transporter 1 (INT1) and H^+^/hexose symporter sugar transport protein 1 (STP1) were down-regulated. The tissue and cellular localization of these differentially expressed transporters should be further investigated to understand its biological role on hexose allocation. Arabidopsis AtSWEET11 from clade III is responsible for sucrose phloem loading in arabidopsis [23], and AtSUT2/SUC3 is inducible by sucrose and located at sieve elements [24, 25]. Up-regulation of these two sugarcane homologs in high biomass sugarcane might reflect the active translocation of sucrose between sink and source tissues. Other than sugar transporters, we also observed altered transcript expression in two phosphate transporters, PHT1;5 and PHT1;7, and an organic cation transporter OCT2. The biological function of these three transporters remains elusive.

### Transcription factors involved in plant growth and secondary cell wall metabolism were regulated

**Fig. 6:**
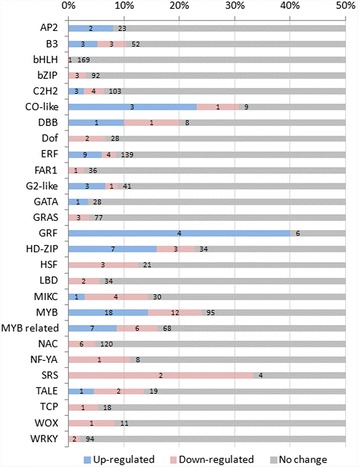
Differentially expressed transcription factors (TFs) between extreme segregated sugarcanes. Up-regulated TF in high biomass sugarcane is indicated as red and down-regulated TFs as blue. TF with no expression difference between two groups is shown in grey. The number of TF in each category is labeled on the bar chart and plotted as accumulative percentage

In addition to direct expression regulation on cell wall metabolism genes, we found that transcription factors (TFs) involved in cell wall biosynthesis and plant growth regulation were also differentially expressed between the two sugarcane groups. In total, 63 and 69 TFs were up- and down-regulated, respectively, in the high biomass group compared to the low biomass group (Fig. 6). We observed a high proportion of growth regulating factor (GRF), SHI-related sequence (SRS), and MYB TF were differentially expressed.

Specifically, four out of 10 GRFs were up-regulated in the high biomass group. In Arabidopsis, GRFs are known to regulate leaf, shoot and root growth and are mainly expressed in growing tissues [26, 27]. Over-expression of AtGRF5 causes increased leaf size with increased number of chloroplast per cell, leading to a higher photosynthetic rate [28]. The up-regulation of these four GRFs in high biomass sugarcane provides candidate regulators to potentially improve biomass by engineering the photosynthetic efficiency.

Over-expressing SRS in *Brassica rapa* and poplar showed suppressed plant growth with shorter stem epidermal cell length [29, 30]. Two out of six SRS TFs in the high biomass group were down-regulated by 4.6 to 10.6 fold (Additional file 2: Table S2). We speculated that these two down-regulated SRSs are also involved in increasing the plant’s growth rate in high biomass sugarcane.

The other notable differentially expressed TF family is MYB, of which multiple members have been previously identified as being involved in secondary cell wall metabolism [31]. To gain insight into the potential roles of these MYB TFs, we built a phylogenetic tree based on sorghum, maize and Arabidopsis MYB protein sequences, and investigated the published functions of maize and Arabidopsis orthologs. There were 18 up-regulated and 12 down-regulated MYB TFs between the two groups. Sobic.008G112200, the ortholog of AtMYB46/83 and ZmMYB46, was expressed 3.5 times higher in the high biomass group. AtMYB46/83 are the master switches of the secondary cell wall metabolite network regulating phenylpropanoid, cellulose, xylan and the lignin pathways [32–34]. Double *myb46/myb83* Arabidopsis mutant plants show arrested plant growth without secondary wall in vessels [33]. ZmMYB46 can restore the secondary cell wall deficiency of these double mutant plants [35], indicating the conserved role of this MYB gene in monocot and eudicot. Furthermore, Sobic.001G358900, Sobic.002G3887700 and Sobic.003G183800, which are homologs of rice cellulose synthase activating OzMYB61 [36], were up-regulated in the high biomass group. Together with the finding of five up-regulated cellulose synthase transcripts in high biomass sugarcane, this finding strongly suggested that the elevated MYB TF and cellulose synthase transcript levels in high biomass sugarcanes facilitate cell wall construction during plant growth.

Interestingly, we observed a paralog (Sobic.001G219200) of sorghum SbMYB60 being up-regulated by five fold in the high biomass group. Over-expression of SbMYB60 in sorghum increases the transcript expression of all but one monolignol biosynthesis genes in leaf, syringyl lignin content and lignin concentration in biomass [37]. On the other hand, Sobic.002G279100, a homolog of maize ZmMYB31/ZmMYB42 that negatively regulates monolignol synthesis and lignin content [38, 39], was also up-regulated in the high biomass group. The biological function and temporal-spatial expression of these two homologs (Sobic.001G219200 and Sobic.002G279100) on primary and secondary cell wall lignin deposition should be further studied in sorghum and sugarcane.

In addition to the MYB transcription factors known to be involved in secondary cell wall metabolism, there were three up-regulated MYB genes in the high biomass group without known function in cell wall metabolism. These three genes are Sobic.004G191800, Sobic.006G107500, and Sobic.007G136500 with 5.3 to 8.5 fold higher transcript expression and are homologous to AtMYB16 and AtMYB106 which regulate leaf cuticle formation [40, 41]. How these three sorghum homologs are related to biomass accumulation should be explored in future studies.

## Discussion

Sugarcane has been selected as one of the most promising species for lignocellulosic biofuel production due to its rapid growth rate and high biomass yield [5, 42, 43]. Breeding for high sucrose content sugarcane has been ongoing for over 100 years, and recent research on sugarcane with high biomass yield has been undertaken [15]. However, the molecular study of the mechanisms regulating these desirable traits has been hindered by the limited availability of genomics resources for sugarcane. Since a reference genome is not available for the complex polyploid genome of sugarcane, we used the well-annotated sorghum genome as reference in this transcriptomic analysis. The biomass-related candidate genes revealed in this study can be immediately transferred to sorghum, another potential biofuel crops, for breeding.

In our RNAseq analysis between high and low biomass sugarcane F2 progenies, it is clearly shown that the genes involved in hexose biosynthesis and degradation are tightly regulated and correlated with biomass accumulation. The fine-tuning of sink and source hexose concentrations through hexose metabolism and transporters allows the plant to allocate the photosynthate for plant growth (i.e. cell expansion and division) and other carbohydrate storage. In the stem, sugarcane mainly stores sucrose rather than carbohydrates, in contrast to other monocots such as rice and maize. The large pool of sucrose stored in stem tissues is a great source to translocate to developing cells and remobilize into glucose, which can then be used for cell wall biosynthesis. The positive correlation between glucose content in stems and total biomass we observed strongly supports this hypothesis. Surprisingly, even when stem glucose level in high biomass sugarcanes is higher, there is no significant decrease in stem sucrose levels (*r* = −0.28, *p* = 0.20), and the stem sucrose content is not related to biomass accumulation. The supply of glucose precursors in the stem, could thus be provided by the breakdown of cell wall polymers, increased photosynthate production or other metabolic pathways [44]. Previous studies also suggested that whole plantlet photosynthesis is highly associated to sugarcane biomass yield [45] and is not limited by the high sucrose content stored at stem through feedback [45, 46].

Regulation of the hexose pool and allocation can support the cell wall metabolism and rapid cell growth, which was demonstrated for this F2 population with high transcript expression of cell wall anabolism and catabolism genes. The high transcript expression of cellulose synthase, laccase, callose synthase, growth regulating factors and cell wall-related MYB transcription factors in the high biomass interspecific F2 progenies provides fundamental working basis for future sugarcane breeding and selection based on these candidate genes. It has been shown that cellulose synthase and cellulose-synthase like gene families expression are related to sugarcane internode maturity [47]. A further study of transcript expression in different stages of internode in high and low biomass individual will provide an in-depth correlation between cell wall metabolism and biomass accumulation. In addition to increasing the cell wall biosynthesis, recycling of cell wall biopolymers by degradation of existing ones can conserve glucose precursors for cell wall reconstruction of new cells. Our transcriptomic study deciphered the tight interaction of carbohydrate allocation between cell wall metabolism and hexose storage, which in turn affects total biomass accumulation. Previous studies demonstrated successful engineering of cell wall contents in potential biofuel crops such as sugarcane [11, 48], sorghum [37, 49], and poplar [50] but some of the candidate genes showed adverse plant growth with reduced biomass [9]. This might be due to the limited hexose available in growing tissues or the withdrawal of hexose precursors from plant development pathways. By including hexose metabolism candidate genes found in this study, energy cane with fast growth rate and high biomass yield could be engineered.

Ideal energy cane should have high dry biomass with low lignin content for better degradation [5]. Our study demonstrated that the selection of extreme segregants from an interspecific *Saccharum* cross proved to be an efficient and simple method to generate sugarcanes with high yields that out-perform their parents. Among the 116 extreme F2 segregants we studied between 2010 and 2012, the highest performing F2 sugarcanes produced 21–217% more total dry mass than one of the parents, *Saccharum officinarum* LA Purple. The worldwide average sugarcane yield during 2010–2012 were 71.4, 70.4, and 70.4 t fresh weight per ha per year. Assuming a moisture content of 76%, the average dry weight yield would be 17.1, 16.9, 16.9 t dry matter per ha per year. During 2010–2012, the highest biomass yield from our F2 population was 98.7, 163.8 and 159.3 t dry matter per ha per year, respectively (Tyler Jones and Chifumi Nagai, unpublished results), suggesting there is a huge amenability of yield improvement using interspecific crosses of sugarcane. Furthermore, with the large number of F2 progenies produced from an interspecific cross, we could easily screen for sugarcane with lower lignin content to improve recalcitrance. The selected energy canes with both desirable traits would be incorporated into energy cane breeding programs immediately to generate high yield sugarcane with enhanced digestibility for biofuel production.

## Conclusions

The transcriptomic analysis between high and low biomass sugarcanes demonstrated that the cell wall and hexose metabolism are pivotal to biomass accumulation. In this study, the up-regulation of a high proportion of cell wall metabolism genes including cellulose synthase, laccase, callose synthase, and pectinesterase was observed in high yield sugarcanes, together with the high transcript expression of growth regulating factor and cell wall-related MYB transcription factors. In addition, UDP-glucose synthesis and sucrose conversion genes were differentially expressed between the two groups. These results revealed the tight relationship between hexose allocation and cell wall biosynthesis during plant growth, and identified the genes contributing to biomass accumulation. All of these findings can be directly applied in traditional breeding and genetic engineering to improve biofuel crops. Furthermore, the development of a population of extreme F2 segregants allows us to immediately incorporate these high performing clones into current energy cane breeding programs. This study enhances our molecular knowledge of sugarcane growth and provides a valuable resource with diverse dry matter yield.

## Additional files

Additional file 1: Table S1. List of differentially expressed genes involved in carbon fixation and Calvin cycle. List of ifferentially expressed genes involved in carbon fixation and Calvin cycle between high and low biomass group. (XLSX 9 kb)

Additional file 2: Table S2. List of differentially expressed transcription factors. List of differentially expressed transcription factors and its log2 fold change. (XLSX 14 kb)

## Abbreviations

DE: Differentially expressed
FDR: False discovery rate
GO: Gene Ontology
GRF: Growth regulating factor
INT1: myo-inositol exporter inositol transporter 1
PCA: Principle component analysis
SRS: SHI-related sequence
STP1: Sugar transport protein 1
STP10: sugar transport protein 10
SuSy: Sucrose synthase
SUT2/SUC3: sucrose transporter 2
